# Obstetrics at Queen Charlotte's Hospital

**Published:** 1908-05-09

**Authors:** 


### Obstetrics.

### OBSTETRICS AT QUEEN CHARLOTTE'S HOSPITAL.

In the very admirable clinical report for 1907 which has lately been issued from the Queen Charlotte's Lying-In Hospital there are several points of interest to those who deal with midwifery cases, whether as specialists or as general practitioners. In certain respects it would be in a high degree misleading to regard the statistics of such a charity as applicable to the community at large. Thus the percentages of contracted pelvis and of difficult labour from other causes are naturally somewhat high in a lying-in hospital, partly because practitioners confronted with grave cases amongst the poorer classes often send their patients at once to hospital, and partly because many women who are known to be the subject of some obstetric disability return in successive pregnancies for admission. But in a good many other respects it is fairly safe to base conclusions as to the trend of modern obstetric practice from these records: for the number of deliveries was 1,700, and if this number is rather small for any sweeping generalisations, it is yet large enough for some very instructive comparisons. Moreover, whether or not the registers have been kept by more than one observer, at least the method has been sufficiently uniform and carefully followed out, if one may judge by the systematic and logical management of the report.

One of the earliest tables shows that, among vertex presentations, but 65.6 per cent. were of the first and second (occipito-anterior) varieties, and of these the former and the latter were respectively as three to one. In 32 per cent. an occipito-posterior presentation rotated forwards, and in 2.4 per cent. it remained posterior. These figures are no doubt influenced to some extent by the undue frequency of contracted pelvis in the Hospital; but even after allowing something for that it would seem as if the third and fourth vertex positions are commoner than is generally believed.

The next point of importance is in connection with the prognosis of breech presentations. Excluding eleven macerated, non-viable, or hydrocephalic fœtuses, and excluding also all cases of podalic version, there were thirty-four infants delivered in this way, only two of which failed to survive. That is to say, the infantile mortality was but 5.8 per cent., which, though doubtless exceptionally low, goes far to support the common suspicion that most text-books take too gloomy a view of the prognosis of this presentation. It is also noteworthy that in this respect the infants of primiparous mothers came off just as well as those of multiparæ: nor can we omit to mention that in seven of the thirty-four cases the pelvis was contracted, and that in these seven cases only one infant succumbed.

From the statistics of placenta prævia and accidental hæmorrhage it seems clear not only that multiparæ are very much more liable than primiparæ, as is well known, to both affections, but also that both are distinctly not rare in the hospital. In this connection it is to be observed that there was no case of completely concealed accidental hæmorrhage, nor was there any one among the eighteen cases in which any reason or explanation could be assigned for the onset of the trouble. One case terminated fatally, and apparently this was the only one treated by plugging the vagina. As to post-partum hæmorrhage all patients losing 20 oz. or more are included in the classification, and they amounted in number to sixty. Three of these were of very extreme degree, one of 50 oz. and two of 60 oz. each; but it is satisfactory to note that no patient died from this cause. The treatment adopted was, in the registrar's words, "to knead the uterus, to give a hot douche, intra-uterine or vaginal according to the severity of the case, and ergot. In a few cases hot saline fluid was injected into the rectum, and strychnine was given. In one case it was found necessary to employ bi-manual compression of the uterus." When it is borne in mind that from the nature of the case this complication may be unduly prone to occur, the results obtained are very satisfactory.

In 383 out of the 1,700 patients was albumin found in catheter specimens of urine; but of these only seven actually suffered from eclampsia, nor was this in any case of a very severe type. Curiously all seven, after good recoveries, left hospital free from albuminuria, whereas seven others who had œdema and albumin without convulsions were still albuminuric on leaving. The very large percentage of patients who passed albumin at the time of delivery is perhaps the most remarkable feature of this part of the report.

There are some valuable remarks also on the subject of the induction of labour, which was adopted for contracted pelvis thirty-six times. The shortest interval between the insertion of a bougie and t[illegible] completion of labour was seven hours, the longe[illegible] ten days; but the average maintained the somewhat surprisingly high figure of seventy-six hours, as in the previous year. The use of bougies of larger diameter than before has shortened the mean duration of induced labour, though two or three exceptionally long cases have kept up the average to the old figures. Labour was also induced by bougies six times for albuminuria, the induction periods being then much shorter than in the contracted pelvis cases. For Cæsarean section the figures are less satisfactory than for induction in contracted pelvis, as two mothers out of seven died.

Lastly, the results of premature birth as affecting the infants are worthy of comment. Thirty premature infants were born dead, 122 alive; of the latter ninety-eight survived and left the hospital, and twenty-four died after delivery. Of these twenty-four not one weighed more than five pounds; while of the ninety-eight, fifty-four were under that weight, and the remaining forty-four between it and seven pounds. The figures speak for themselves of the success with which these babies are treated, though it would perhaps be instructive if their subsequent career could be followed.

